# Molecular and Phenotypic Profiling for Precision Medicine in Pancreatic Cancer: Current Advances and Future Perspectives

**DOI:** 10.3389/fonc.2021.682872

**Published:** 2021-06-23

**Authors:** Koji Miyabayashi, Hayato Nakagawa, Kazuhiko Koike

**Affiliations:** Department of Gastroenterology, Graduate School of Medicine, The University of Tokyo, Tokyo, Japan

**Keywords:** precision medicine, patient derived organoid, patient derived xenograft, liquid biopsy, molecular subtypes

## Abstract

Pancreatic cancer is the most common lethal malignancy, with little improvement in patient outcomes over the decades. The development of early detection methods and effective therapeutic strategies are needed to improve the prognosis of patients with this disease. Recent advances in cancer genomics have revealed the genetic landscape of pancreatic cancer, and clinical trials are currently being conducted to match the treatment to underlying mutations. Liquid biopsy-based diagnosis is a promising method to start personalized treatment. In addition to genome-based medicine, personalized models have been studied as a tool to test candidate drugs to select the most efficacious treatment. The innovative three-dimensional organoid culture platform, as well as patient-derived xenografts can be used to conduct genomic and functional studies to enable personalized treatment approaches. Combining genome-based medicine with drug screening based on personalized models may fulfill the promise of precision medicine for pancreatic cancer.

## Introduction

Pancreatic ductal adenocarcinoma (PDAC) is one of the most lethal malignancies, with an average 5-year survival rate of less than 10% (1). More than half of patients are diagnosed with metastatic disease, which is associated with a 5-year survival rate of only 3% (1). Early detection methods and effective therapies need to be developed to improve the prognosis of PDAC (2). The recent revolutionary improvement in genetic analysis technology offers the promise of using genetic information for personalized medicine. In pancreatic cancer, a number of studies have described a genetic background characterized by a set of commonly mutated genes in core molecular pathways and significant intratumoral heterogeneity. Resistance to chemotherapeutic agents has also been attributed to difficulties in drug delivery through a rich stromal microenvironment, as well as the nature of the cancer itself. For these reasons, the development of therapeutics for pancreatic cancer has been challenging, and many promising drugs have failed in clinical trials.

**Figure 1:**
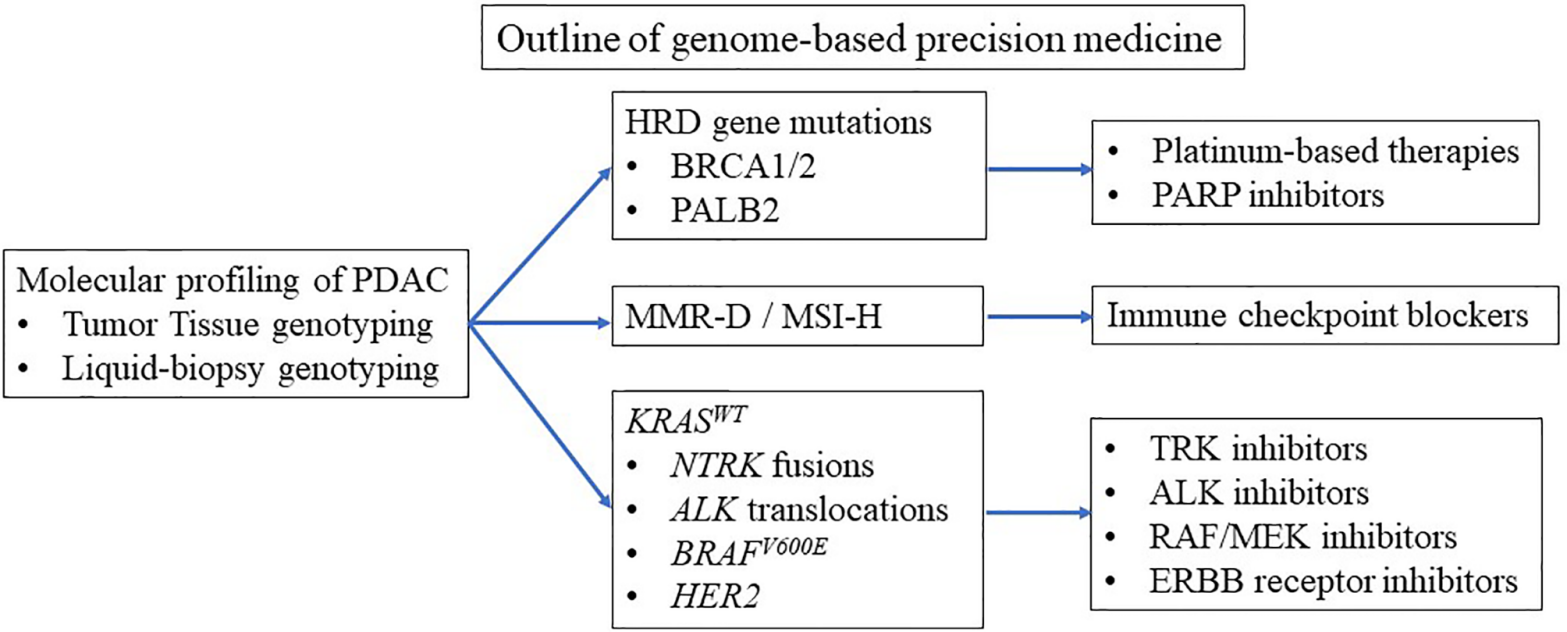
Outline of genome-based precision medicine.

Clinical trials are currently underway to tailor treatment to underlying mutations (3–5). Basically, three groups of pancreatic cancer patients benefit from personalized medicine (**Table 1** and **Figure 1**). Patients with *BRCA1* and *BRCA2* mutations benefit from platinum-based therapy and poly (ADP-ribose) polymerase (PARP) inhibitors (3, 4, 6–9). Patients with microsatellite instability-high (MSI-H) benefit from immune checkpoint blockade (ICB) therapy (5, 10). Patients with wild-type *KRAS* (*KRAS^WT^*) often carry other oncogenic mutations such as *BRAF* (3, 4), which can be candidates for small-molecule therapy. To enroll patients in genome-based precision medicine, recent reports have suggested that diagnosis by liquid biopsy is promising (11). However, the number of patients who can benefit from precision medicine is limited due to the limited number of mutations leading to precision medicine (3, 4).

**Table 1:** Ongoing clinical trials.

Homologous reconbination deficiency (HRD) related therapies			
Targets	Patients	Drugs	Trials
HRD genes	metastatic PDAC with germline/somatic *BRCA, PALB2* mutations	Rucaparib	Phase 2 NCT03140670
	solid tumors with germline/somatic DDR gene mutations	Rucaparib	Phase 2 NCT041717000
	metastatic PDAC with DDR gene mutations	Rucaparib	Phase 2 NCT03337087
	metastatic PDAC with DDR gene mutations	Rucaparib	Phase 2 NCT02890355
	advanced PDAC with *BRCA1/2, PALB2, CHEK2 or ATM* mutations	Niraparib	Phase 2 NCT03601923, Phase 2 NCT03553004
	metastatic PDAC with DDR gene mutations	Niraparib	Phase 1b/2 NCT03404960
**Mismatch repair deficiency (MMR-D) or microsatellite instability high (MSI-H)**			
**Targets**	**Patients**	**Drugs**	**Trials**
PD-1	advanced/metastatic PDAC	Pembrolizumab	Phase 2 NCT04058964, Phase 2 NCT03331562, Phase 2 NCT03264404, Phase 2b NCT02907099, Phase 1 (Part B) NCT04007744
	advanced/metastatic PDAC	Nivolumab	Phase 2 NCT03697564
***KRAS wild-type***			
**Targets**	**Patients**	**Drugs**	**Trials**
*NTRK* fusion	advanced/metastatic solid tumors with *NTRK/ROS1/ALK* gene rearrangements	Entrectinib	Phase 2 NCT02568267
*ALK, ROS1 *gene translocations	solid tumors with *ALK, ROS1* translocations	Crizotinib	Phase 2 NCT02465060(MATCH screening trial) Phase 2 NCT02465060(MATCH screening trial)
*BRAF^V600E^*	solid tumors with *BRAF^V600E/R/K/D^*	Dabrafenib	Phase 2 NCT02465060(MATCH screening trial)
*HER2*	advanced PDAC, biliary cancers	Afatinib	Phase 1b NCT02451553
	solid tumors with *NRG1* fusion	Zenocutuzumab	Phase 1/2 NCT02912949

The recent identification of two major transcriptional subtypes of PDAC with characteristic histopathological features and different prognoses has provided a new perspective for developing therapies (12–15). These include a “basal-like” (or squamous) subtype, which is poorly differentiated and carries a worse prognosis, and a “classical” (or progenitor) subtype, which is well differentiated and has a better prognosis (13, 14, 16). Basal-like and classical subtypes can predict the response to chemotherapy (12, 17–19) and are associated with stromal subtypes. Stroma-targeted therapies have largely failed because of their complicated features and models which recapitulate the tumor microenvironment (TME), and drug responses to stroma-targeted therapies are needed. In addition to precision medicine based on molecular profiling, phenotypic profiling, such as drug screening using personalized models, is useful in the clinic. The patient-derived xenograft (PDX) has been established as a preclinical tool to improve drug screening and development; however, the PDX model requires sufficient tissue for transplantation, and failures are not uncommon (20–23). A recently described, organoid culture system can be exploited for molecular and phenotypic profiling to enable personalized therapeutics (24, 25). A variety of approaches using co-culture of organoids with stromal cells have been established and used for ICB therapy testing. Organoid technology may bridge the gap between cancer genetics and clinical trials, enabling personalized therapy.

Several studies have described the usefulness of precision medicine based on molecular profiling (3–5) and phenotypic profiling (24, 25). Approaches using both genome-based medicine and individualized model-based drug screening will be useful for achieving precision medicine for pancreatic cancer.

## Molecular Subtypes of Pancreatic Cancer

### Genomic Subtypes

Recent genomic analyses have revealed the mutational landscape of PDAC (14, 26–28). More than 90% of PDAC harbor activating KRAS mutations. Mutations in KRAS are seen in all stages of pancreatic intraepithelial neoplasia (PanIN). The commonly accepted model of carcinogenesis describes a stepwise progression from normal pancreatic epithelium to PanIN and finally to adenocarcinoma due to accumulation of genetic alterations. Inactivation of tumor suppressor genes, such as *TP53*, SMAD family member 4, and cyclin-dependent kinase inhibitor 2A, is seen with progressive PanIN development and occurs in more than 50% (29, 30). The prevalence of recurrently mutated genes then decreases to ~10%, which aggregates into core molecular pathways, including KRAS, wingless and int (WNT), NOTCH, DNA damage repair, RNA processing, cell cycle regulation, transforming growth factor beta (TGF-β) signaling, switch/sucrose non-fermentable, chromatin regulation, and axonal guidance (14, 26–28). Pancreatic tumors exhibit a high frequency of chromosomal rearrangement (31), and a subset of PDAC tumors may progress *via* chromosomal rearrangements instead of stepwise progression *via* accumulation of genetic mutations (31). Chromosomal rearrangements and amplification of *KRAS* are reportedly linked to poor outcomes in PDAC patients (32).

Pathway analyses based on genetic changes have detected associations of various pathways with outcome in PDAC patients. DNA repair-associated pathways are associated with a poor prognosis, whereas beta-catenin signaling is associated with improved outcomes (33). Many of these pathways can be actionable therapeutic targets in preclinical models and in the clinic. Molecular profiling suggests that up to 25% (range 12–25%) of pancreatic cancers harbor actionable molecular changes (5). Three main groups, such as genetic changes in homologous recombination deficiency (HRD), mismatch repair deficiency (MMR-D)/high microsatellite instability (MSI-H), and oncogene alterations, such as *BRAF* mutation and *NTRK* gene fusions in *KRAS^WT^*, are considered potential actionable mutations. In the American Society of Clinical Oncology (ASCO) guidelines, early testing for actionable genomic changes (both germline and somatic) is recommended for pancreatic cancer patients who are likely to be potential candidates for additional treatment after first-line therapy (34). Patients with *BRCA* mutations, MMR-D/MSI-H, and *NTRK* gene fusions can be given tailored therapies, such as PARP inhibitors, ICB therapy, and TRK fusion inhibitors, respectively. A retrospective analysis of the Know Your Tumor program testing matched therapies following molecular profiling revealed significantly longer overall survival (OS) after PARP inhibitor therapy in patient with *BRCA* mutations or after ICB therapy in those with MMR-D compared with patients who received unmatched therapies (2.58 *vs.* 1.51 years) or those without an actionable molecular change (2.58 *vs.* 1.32 years) (3). In this study, the most common actionable alteration was mutations in the DNA damage response (DDR) pathway, including *BRCA* mutations. These data suggest promise for this personalized approach.

#### HRD

Diverse defects in HR DNA repair genes, such as germline mutations in *BRCA1*, *BRCA2*, and *PALB2*, somatic mutations in *BRCA1* and *BRCA2*, and promoter methylation of *BRCA1*, have been reported in breast and ovarian cancers (35, 36). *BRCA* mutations are also associated with an increased risk for pancreatic cancer, and 4% to 7% of patients with pancreatic cancer have a germline *BRCA* mutation (8). *BRCA* genes encode for proteins involved in the HR repair of DNA double-stranded breaks. Cells with deficient HR repair are sensitive to PARP inhibition. PARP enzymes are key components in the repair of DNA single-stranded breaks and replication fork damage (37). PARP inhibition causes accumulation of such lesions through catalytic inhibition and trapping of PARP on DNA at the sites of single-stranded breaks. These processes eventually result in double-stranded breaks, which cannot be accurately repaired in tumors with HRD. Thus, PARP inhibitors cause accumulation of DNA damage and tumor-cell death. Accordingly, PARP inhibitors are selectively effective for cells with HRD due to *BRCA1* or *BRCA2* mutations (38, 39).

Recent investigations of genomic profiling in large cohorts of PDAC have reported the significance of HRD in predicting sensitivity to platinum-based therapy and PARP inhibitors (3, 4, 6, 7). According to ASCO guidelines, treatment with platinum-based chemotherapy or the PARP inhibitor olaparib is recommended for patients who have a germline *BRCA1* or *BRCA2* mutation. A recent randomized phase III trial (POLO) demonstrated the efficacy of olaparib, a PARP inhibitor, in germline *BRCA*-mutated metastatic PDAC (8). Among the 154 enrolled patients, progression-free survival (PFS) was significantly longer in the olaparib group than the placebo group (7.4 *vs.* 3.8 months). Furthermore, another recent randomized phase II trial showed that patients with germline *BRCA1/2*- or *PALB2*-mutated PDAC benefit from first-line platinum chemotherapy, with median OS and PFS of 15.5 (14.6–19) and 7 (6.1–8.1) months, respectively. Patients with HRD had improved PFS compared with no HRD when treated with first-line platinum therapy but not with first-line non-platinum therapy (9). These results suggest that HRD can effectively be targeted in PDAC.

In addition to mutations in canonical HR genes, a comprehensive evaluation of HR gene mutations is needed beyond germline *BRCA* mutations to understand their sensitivity to DDR-targeted therapies, including platinum-based therapy. Multiple groups have identified a broader group of patients with HRD sensitive to DDR-targeted therapies (3, 4, 7). The concept of “BRCAness” was introduced to describe the clinical and biological features in some sporadic tumors shared with tumors harboring germline BRCA1/2 mutations. Polak et al. (40) explored signature 3, a mutational signature prevalent in tumors with BRCAness, and found altered expression of *PALB2* and *RAD51*, which are genes that are important in the HRR pathway. In addition, signature 3 has been found in tumors with both germline and somatic *BRCA1*/*2* mutations (40). Thus, signature 3 can be considered a potential biomarker that could lead to BRCAness-targeting therapies. O’Reilly and colleagues (7) evaluated the mutational status of HR genes and HRD genetic signatures to determine their benefit to platinum therapy. They observed that patients with HRD had significantly improved PFS when treated with first-line platinum-based therapy compared with those who received first-line non-platinum-based therapy. Subgroup analyses suggest that patients with either pathogenic somatic or germline *BRCA1*, *BRCA2*, or *PALB2* mutations, as well as biallelic loss of other rarer HR genes, such as *ATM* and *CHEK2*, could be recommended for platinum-based therapy. Aguirre and colleagues (4) observed four samples that did not have clear DNA changes or mRNA downregulation of *BRCA1*, *BRCA2*, *PALB2*, or *RAD51C* but nevertheless had enrichment of HRD/signature 3 compared with samples with HRD. These data suggest that signature 3 can be recommended for platinum-based therapy and PARP inhibitors. Furthermore, patients with biallelic HRD show higher tumor mutation burden (TMB), indicating the potential benefit from immunotherapy as shown in other types of cancer (41, 42).

#### MMR-D/MSI-H

Immune checkpoint inhibitors have been an effective therapy for MMR-D/MSI-H cancers regardless of tumor type, although activity may vary by tumor type. MMR-D occurs as a consequence of loss-of-function changes in MMR genes (*MLH1, PMS2, MSH2, MSH6*) because of the inherited germline mutations known as Lynch syndrome, or because of the biallelic somatic inactivation of MMR genes. In PDAC, approximately 1% of patients have MMR-D or MSI-H due to Lynch syndrome or somatic MMR gene mutations (43, 44). The normal MMR system can correct the process of DNA replication errors but MMR-D results in an inappropriate response to DNA mismatches, increasing the possibility of gene mutation. MMR-D causes MSI-H by missing or inserting one or more of the repeating units in the inappropriate process of DNA replication and repair. MMR-D and MSI-H are generally associated with high TMB. A high TMB increases the potential number of neoantigens, and these neoantigens can be presented by the tumor cell and recognized by host immune cells, which are also known as tumor-infiltrating lymphocytes (TILs) that migrate into TME (45, 46). TILs, particularly CD8+ cytotoxic T cells, orchestrate a significant antitumor response to eliminate tumor cells (45, 46). Detection of MMR-D and/or MSI-H was proposed as a biomarker of an immunogenic tumor and response to ICB therapy, such as anti-programmed cell death protein 1 (PD-1) inhibition. The immune checkpoint inhibitor pembrolizumab is currently approved for treatment of MMR-D/MSI-H cancer regardless of the histology. In ASCO guidelines, pembrolizumab is recommended as a second-line therapy for PDAC patients with MMR-D or MSI-H (34).

The recently published KEYNOTE 158 study (10) and NCI-MATCH study (5), which investigated the efficacy of ICB therapy in non-colorectal cancers, clearly suggested that MMR-D in solid tumors is a predictor of the response to ICB therapy. However, ICB therapy has low efficacy against pancreatic cancer, suggesting that cancer type-specific responses show variable clinical outcomes, and that disease-specific biological factors may have an independent impact on ICB response, regardless of MMR-D status. Regarding the underlying mechanisms, the degree of T-cell infiltration is critical for predicting the efficacy of ICB therapy in other types of cancers (47–52), and a small subset of patients with MSI-H tumors exhibit T-cell infiltration and sensitivity to immunotherapy (53). In pancreatic cancer, dense stroma with desmoplastic reaction may function as a physical barrier and affect the infiltration of myeloid-derived suppressor cells and T cells in tumor stroma (54, 55). In addition, PDAC exhibits substantial immunological heterogeneity, with tumors influencing T-cell infiltration (45, 56–58). For example, Stanger et al. (46) observed heterogeneity in the degree of T-cell infiltration in their cohort of 12 PDAC patients (none of whom were MSI-H), in line with prior reports for PDAC (45). The abundance of PD-1+CD8+ T cells was more predictive of immunotherapy response than was total CD8+ T cell infiltration alone. Furthermore, Leach and colleagues (59) identified mucin 16 neoantigens as T-cell targets in PDAC and as potential biomarkers of immunogenic tumors that may guide the application of immunotherapies (59). These results suggest that both the quality and quantity of tumor-infiltrating CD8+ T cells are critical for predicting the immunotherapy response, and novel biomarkers are needed to predict the status of tumor-infiltrating CD8+ T cells.

Furthermore, the abovementioned results suggest that MMR-D status is not a perfect predictor of immunotherapy response. Loss-of-function changes in MMR genes can sometimes be a passenger mutation/change and responses to immune checkpoint inhibitor therapy could be affected by founder mutations that determine the molecular behavior of cancer (60). MSI-H and high TMB may be a better predictor of the immune checkpoint inhibitor response, as these markers are highly associated with MMR-D-driven carcinogenesis (61). The basis for these differences in T-cell infiltration is poorly understood in PDAC, where most tumors share the same oncogenic mutations. Further prospective studies are needed to evaluate the predictor role of characterization of T-cell infiltration on ICB response/resistance in cancer patients with MMR-D tumors.

#### KRAS^WT^

*KRAS* mutation is a major driver mutation in pancreatic cancer and more than 90% of pancreatic cancer patients harbor *KRAS* mutation. Recently drugs targeting *KRAS^G12C^* are available and clinical trials suggested promising results (62–64). However, *KRAS^G12D^* and *KRAS^G12V^* mutations are more common in pancreatic cancer, and these mutations are still undruggable. In *KRAS^WT^* PDAC patients, *NTRK* fusions*, ALK* rearrangements, *ROS, NRG1* rearrangements*, BRAF, PIK3CA*, and a number of cancer-associated genes representing potential drivers have been identified (e.g., *ERBB2, STK11, GNAS, CHEK2*, and *RB1*), as potential targets.

Gene fusions involving *NTRK1, NTRK2*, or *NTRK3* (*TRK* fusions) are found in many pediatric and adult malignancies (65). *NTRK* fusions are rare in PDAC and are identified in less than 1% of tumors (66). Pishvaian et al. (67) reported a partial response to entrectinib, a potent TRK and ROS1 inhibitor in a subgroup of advanced PDAC patients. Larotrectinib, a highly selective small-molecule inhibitor of the TRK kinases, has shown efficacy in preclinical models and in patients with tumors harboring TRK fusions. In ASCO guidelines, in patients with tumors harboring *NTRK* fusions, treatment with larotrectinib or entrectinib is recommended as treatment options after first-line therapy such as FOLFIRINOX and gemcitabine plus nab-paclitaxel (GnP) (34). *NRG1* rearrangement contributes to susceptibility to ERBB inhibitors and anti-EGFR antibodies and clinical trials are ongoing (68, 69). *ALK* gene translocations have been reported in 0.16% of PDAC, and crizotinib is reportedly effective for a PDAC patient with *ALK* gene translocation (70).

*BRAF^V600E^* mutations occurred at a frequency of 3% and were mutually exclusive with *KRAS* mutations. *BRAF^V600E^* could be a driver event based on mouse models (71). Analyses of PDAC cells revealed that *BRAF^V600E^* cells are sensitive to the FDA-approved BRAF inhibitor PLX-4032, while cells with *KRAS* mutations are resistant (33). These data suggest that a subset of patients may benefit from targeted therapy along the KRAS/BRAF axis. Aguirre et al. (4) reported the first therapeutic experience with mitogen-activated protein kinase (MAPK) inhibition in a patient harboring a *BRAF* in-frame deletion. The patient had a partial response to the MEK inhibitor trametinib. A second patient with rapidly progressive disease harboring a *BRAF* mutation was also treated with trametinib but failed to show a response. This heterogeneity in resistance mechanisms will require effective combination treatments with MAPK inhibitors. The Cancer Genome Atlas Research Network reported that the *KRAS^WT^* tumors had significantly elevated tuberous sclerosis complex/mammalian target of rapamycin (TSC/mTOR) signaling pathway activity compared with *KRAS* mutant tumors, indicating that functional activation of the mTOR signaling pathway may be an alternative oncogenic driver in *KRAS^WT^* pancreatic cancer (15).

These data suggest that larger multicenter clinical trials are needed to fully investigate the therapeutic efficacy of the inhibition of upstream and downstream signaling of RAS in *KRAS^WT^* patients with other oncogenic mutations.

#### Liquid Biopsy

Although genome-based precision medicine, such as platinum-based therapy and PARP inhibition, in PDAC patients with HRD is promising, tissue-based genomic sequencing for first-line treatment decision making in PDAC remains challenging due to the turn-around time of obtaining sequencing results, which is 3 to 6 weeks. To enroll patients in genome-based precision medicine, recent reports have suggested that diagnosis by liquid biopsy with a short turn-around time has emerged. Liquid biopsy includes analyses of tumor materials obtained in a minimally invasive or noninvasive manner by collecting blood or other body fluids. Liquid biopsy samples are obtained from saliva, stool, or urine. They include circulating tumor cells (CTCs), circulating tumor DNA (ctDNA), extracellular vesicles, cell-free DNA, and microRNA. More recently, next-generation sequencing-based methods have enabled ctDNA profiling as an alternative to tumor tissue sequencing (72, 73). For example, the TARGET study recently reported the screening of 100 patients using ctDNA sequencing for trial enrollment (74). Most recently, Yoshino and colleagues (11) reported that ctDNA genotyping significantly shortened the screening duration (11 *vs.* 33 days; P < 0.0001) and improved the trial enrollment rate (9.5 *vs.* 4.1%; P < 0.0001) compared with tumor tissue sequencing. Overall, ctDNA was detected in 91.4% (1,438/1,573) of patients; however, the ctDNA detection rate of PDAC was the lowest (83.4% (304/363) compared with other types of cancers, such as esophageal squamous-cell carcinoma cancer (99.1% (107/108) and CRC (96.0%, 628/654). Overall, they detected multiple biomarkers relevant to the selection of treatment, including *KRAS, NRAS, BRAF*, and *PIK3CA* mutations; *ERBB2, FGFR1–2*, and *MET* amplifications; *FGFR2–3, ALK, NTRK1*, and *RET* fusions; and MSI. Liquid biopsy also enables the collection of repeated samples during the course of the treatment of patients and the collection of clones resistant to ongoing therapy. Thus, this technology has the potential to promote innovation in precision medicine.

### Transcriptomic Subtypes

#### Cancer Cell Subtypes

Targeting drugs, according to tumor subtypes, have improved treatment outcomes in other cancers. Identification of therapeutic molecular subtypes in PDAC has been challenging. In addition to genomic subtypes, transcriptomic subtypes have been evaluated to understand the biology of pancreatic cancer. The recent identification of PDAC transcriptional subtypes has provided a new perspective relevant to the development of therapies. These include basal-like/squamous and classical/progenitor (hereafter referred to as basal-like and classical, respectively) (12–14, 75–77). Basal-like tumors are associated with poor outcomes and treatment resistance (12–14, 16–19, 75–77). Two independent clinical trials revealed that basal-like tumors are resistant to FOLFIRINOX-based regimens (19, 78). In those studies, RNA *in situ* hybridization or immunohistochemical analysis of GATA-binding protein 6 (GATA6) was used to differentiate basal-like and classical tumors. The resistance of basal-like tumors to FOLFIRINOX is supported by a recent report by Tiriac et al. (24), who showed that patient-derived organoid (PDO) chemotherapy signatures may predict treatment response. The signatures representing individual cytotoxic agents were applied to the COMPASS cohort, suggesting that basal-like tumors are most likely to have a non–oxaliplatin-sensitive signature (24). To apply molecular subtyping in treatment decision-making for PDAC patients, Rashid et al. (79) revealed that the tumor-intrinsic two-subtype schema of Moffitt et al. is the most replicable, and they developed the Purity Independent Subtyping of Tumors, a clinically usable single-sample classifier based on gene expression data obtained using multiple platforms, including microarrays, RNA sequencing, and NanoString.

The development of subtype-based therapies remains challenging because the genetic and epigenetic aberrations that promote the stable or dynamic regulation of subtypes are unknown. The basal-like subtype consists of small subgroups that are regulated by different mechanisms (14). The master regulators of the basal-like subtype have been identified, and the basal-like subtype is associated with the activation of genes involved in the epithelial–mesenchymal transition, activation of transcription factors such as MYC and TP63, and downregulation of markers for endoderm such as *HNF4A* and *GATA6* (12–14). In addition, expression of the ΔN isoform of TP63 (ΔNp63) and GLI2 promotes the basal-like identity in PDAC (80, 81). Several epigenetic regulator genes, including *KDM6A*, *KMT2C*, and *KMT2D*, are associated with the basal-like subtype (12, 14, 82). Mueller et al. (83) divided the basal-like subtype into the TP63-related transcriptional program with squamous differentiation and the RAS/epithelial–mesenchymal transition-related transcriptional program with undifferentiated cancers. Further characterization of the master regulators of molecular subtypes may lead to the identification of biomarkers and targets for tailored therapies.

Because *KRAS* is the most commonly mutated gene in PDAC, the association between KRAS addiction (KRAS dependency) and a molecular subtype has been debated. KRAS-addicted cells have been previously observed as more classical and epithelial in monolayer cell cultures (12). Collisson et al. (12) showed that classical PDAC cells are relatively more dependent on KRAS and more sensitive to erlotinib than basal-like PDAC cells. Conversely, basal-like PDAC lines are more sensitive to gemcitabine than classical PDAC (12). KRAS ablation induces a basal-like phenotype in surviving cells *in vivo* (80). A study of the inducible KrasG12D;Trp53-/- PDAC mouse model (84) revealed cancer cell-intrinsic mechanisms enabling bypass of KRAS dependency and tumor recurrence (85). Specifically, Yap1 amplification and overexpression enabled escape in approximately one-third of KRAS-negative recurrent PDAC tumors (85) and serves a similar role in lung cancer (86). However, allelic imbalance and elevated expression of mutant KRAS have been associated with aggressive and undifferentiated histological phenotypes in PDAC (32, 83). Furthermore, increased dosage of mutant KRAS is sufficient to induce basal-like features (32, 87). These results suggest that mutant *KRAS* plays an important role in oncogenesis in PDAC, but other epigenetic or microenvironmental factors are critical in regulating molecular phenotypes.

#### Stromal Subtypes

Pancreatic cancer is characterized histologically by a dense stromal reaction with desmoplasia, which creates a physical barrier around the tumor cells and prevents appropriate vascularization and delivery of chemotherapeutic agents (88). The surrounding desmoplasia was formerly considered to promote cancer, and a number of clinical trials targeting the stroma have been conducted to prove this. However, most of those trials failed, and the current understanding is that the stroma is multi-faceted (89–91). To reveal the heterogeneity of stromal components, studies based on single-cell RNA sequencing have been conducted (92–95). Cancer-associated fibroblasts (CAFs) play an important role in the TME, and cancer-derived IL-1 or TGF-β can stimulate the differentiation of surrounding fibroblasts into inflammatory and myofibroblastic CAFs, respectively (93). IL-6 secreted by inflammatory CAFs promote proliferation of the tumor, whereas myofibroblastic CAFs produce the surrounding stroma. Because cancer cells create an environment favorable to themselves, these stromal subtypes are linked to the cancer cell subtypes mentioned above. Mauer et al. (77) reported CAF subtypes using laser capture microdissection and RNA sequencing of pathologically verified PDAC epithelia and their adjacent stroma. The authors detected two subtypes reflecting ECM deposition and remodeling (ECM-rich) versus immune-related processes (immune-rich). There was a strong association between ECM-rich stroma and basal-like tumors, whereas immune-rich stroma occurred more often in association with classical tumors (77, 96). As such, the epithelial and stromal subtypes were partially linked, suggesting potential biomarkers for stroma-targeted therapies in PDAC.

As mentioned previously, TILs are associated with the response to immune checkpoint inhibitors. Therapeutic strategies targeting immune modulators have emerged. Bailey et al. (14) identified an immunogenic cancer subtype, which shares many of the characteristics of classical tumors but is uniquely associated with significant immune-cell infiltration. This cancer cell subtype, as well as Mauer et al.’s immune-rich subtype, has potential as a biomarker for immune therapy. Furthermore, studies using mouse models have revealed potential targets such as colony-stimulating factor 1 receptor, cytotoxic T-lymphocyte-associated protein 4 (97, 98), and CXC chemokine receptor 2 (99, 100), which led to clinical trials. However, due to their complexity, TME-targeted therapies have largely failed (101, 102). Clinical trials of such therapies have been reviewed recently (103). Further investigations are warranted to discover effective TME-targeted therapies.

## Patient-Derived Models

### PDO

Organoids are three-dimensional structures that are grown *in vitro* and recapitulate many aspects of corresponding organs *in vivo*, providing many novel human cancer models. Theoretically, PDOs allow expansion of small tumor samples, enabling the analyses of cancer at any stage. Various human carcinomas have been established from resected specimens and biopsy samples (24, 104–116). Pancreatic tumors contain abundant stromal components and exhibit low neoplastic cellularity, which contribute to the low accuracy of genetic and transcriptional analyses of the neoplastic compartment in bulk tumor tissues. In organoid culture, only the epithelial component expands, thus providing high-quality research materials (24).

**Figure 2:**
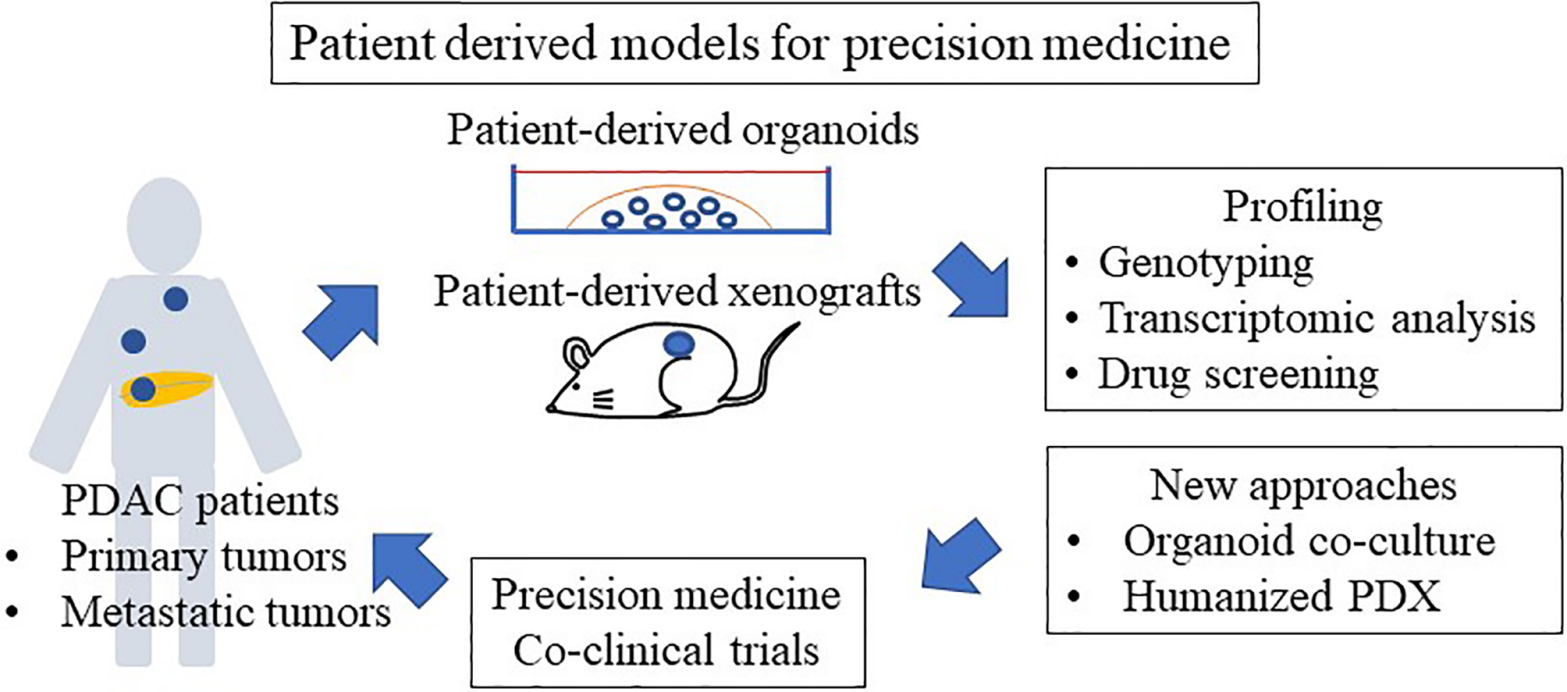
Patient-derived models for precision medicine.

The organoid culture system is a powerful tool for personalized medicine and is used in co-clinical trials because the response of PDOs to drugs largely mimics the initial response of corresponding patients to the same drugs (24, 108, 111, 117, 118) (**Figure 2**). Tiriac et al. (24) established a biobank of 66 pancreatic cancer PDOs, from both biopsy samples and resected specimens, and compared the gene expression of those PDOs with responses to standard cytotoxic drugs and identified transcriptional gene signatures of responders to different chemotherapies. They found that the transcriptional gene signature reflects a drug response in an independent cohort of PDAC patients. Currently, clinical trials using PDOs are ongoing, and PDO can be used to select second-line or adjuvant treatments because the time required to generate and test PDOs is about 4–6 weeks (25). Pancreatic cancer frequently acquires resistance to chemotherapy. Tiriac et al. (24) reported their experience with longitudinal collection of organoids from the same patient undergoing chemotherapy. Interestingly, an organoid collected before the corresponding patient acquired resistance to FOLFIRINOX and gemcitabine/nab-paclitaxel regimens was sensitive to gemcitabine, paclitaxel, 5-FU, and oxaliplatin, whereas organoids collected after the chemoresistance developed were resistant to those chemotherapeutic agents. In addition, the first organoid collected was resistant to mTOR inhibition, whereas subsequent organoids were sensitive to mTOR inhibition. These results suggest that collecting organoids during chemotherapy enables drug selection according to chemosensitivity in PDAC patients. Collecting organoids from metastatic and recurrent sites is difficult. However, Gao et al. (119) demonstrated the feasibility of growing organoids from CTCs from a prostate cancer patient to overcome this shortcoming.

As mentioned, pancreatic cancer is characterized by a dense stromal reaction with desmoplasia. To mimic the tumor environment, co-culture systems have been developed. The co-culture system of pancreatic stellate cells, a resident mesenchymal cell, with pancreatic cancer PDOs, has been established (92). This system enables to produce the desmoplastic stroma and led to the specification of pancreatic CAF subtypes, including inflammatory CAFs and myofibroblastic CAFs (93). Several approaches of co-culturing PDOs with immune and fibroblastic components have been established to predict the efficacy of immune checkpoint inhibitors in other types of cancer (120) (121), which can be applicable to PDOs in PDAC. As with the PDX mouse model, organoid transplant mouse models are a powerful tool for drug screening and biological research. Boj et al. (122) reported that orthotopic transplantation of organoids led to the development of all stages of disease progression including early PanIN, late PanIN, invasive ductal adenocarcinoma, and metastasis. A recent study reported the usefulness of an intraductal transplantation mouse model of PDOs (87), which models the progressive switching of molecular subtypes. These models are promising tools to evaluate human PDAC at any stage to understand its fundamental biology and to identify biomarkers of early disease as well as biomarkers of subtype switching at later stages, contributing to discovery of novel therapeutic strategies.

### PDX

PDXs have emerged as an important platform to discover novel therapeutic strategies and biomarkers (21, 22, 123). PDX models retain key features of donor tumors both histologically and biologically, and effectively recapitulate the chemosensitivity of corresponding patients compared with conventional two-dimensional cell-line-based xenograft models (124–127). Analyses of genetic profiles show good concordance between primary tumors and the tumors derived from PDX models, although there were differences in genes involved in the stromal and immune compartments due to the replacement of the human stroma by murine elements. The key characteristics and practical applications of PDXs can be found in recent reviews (20–23).

PDX models of PDAC patients have been reported (128–130), and one study found a good correlation between response to gemcitabine in PDXs and in PDAC patients (131). The drug response of PDX models remains stable across generations (up to 10 passages) (128, 132). Hidalgo et al. (133) performed an empirical treatment of PDX models with a panel of drugs while the patients were receiving first-line therapy and showed that GnP is effective in PDX models, which is correlated with the efficacy of this combination in the clinic (134). Similarly, lack of efficacy in preclinical studies with PDX predicted failure of the same therapies in the clinic, such as the SRC inhibitor saracatinib and the mTOR inhibitor sirolimus in PDAC (124, 135). Based on these data, PDX models are an essential part of the preclinical screening for new chemotherapeutic agents (**Figure 2**).

PDX models are also used in co-clinical trials, in which they are developed from patients enrolled in clinical trials and treated with the same experimental agents (**Figure 2**). These models are used to evaluate the clinical response based on appropriate endpoints such as response rate or tumor growth delay. PDX models are also powerful tools for simulating tolerance after exposure to therapies used in the clinical setting and to develop strategies for overcoming resistance (136, 137). Furthermore, biological and genetic comparisons between sensitive and resistant models could lead to the discovery of biomarkers of drug efficacy as well as biomarkers for inclusion in clinical studies. In PDAC, PDX studies using gemcitabine revealed expression of deoxycytidine kinase, the gemcitabine-activating enzyme, as a predictor of drug efficacy (128, 138). Similarly, PDX models have been used to determine metabolic and imaging biomarkers (139, 140). This strategy provides an interesting platform to evaluate drug response in the patients and PDX models simultaneously, and to investigate biomarkers of sensitivity and resistance, as well as new combination strategies to overcome emerging resistance pathways. These findings suggest that PDX models hold promise for precision medicine in PDAC.

Regarding the drawbacks, in most patients, obtaining individualized PDXs to guide treatment is not feasible because of the low success rate of engraftment, the discrepancy between the time needed for PDX expansion and treatment, and the rapidity of disease progression in patients (131, 133). PDX models are generally established from surgical specimens, which provide a large amount of tumor tissue. However, because most PDAC patients are inoperable, generating PDX from smaller samples, such as fine-needle aspiration for personalized therapy, is more useful. To resolve this problem, CTC-derived xenografts are now applied to evaluate other types of cancer such as breast cancer (141), prostate cancer (142), gastric cancer (143), small-cell lung cancer (SCLC) (144), and melanoma (145). A major obstacle is that PDX models require the use of immunocompromised mice, which prevents the evaluation of immunomodulators, such as vaccines, anti-PD-1, and anti-cluster of differentiation 40 (CD40) antibodies. Humanized mice with human immune system in which selected immune components have been introduced may solve the problems. However, human tumor stroma in the cancer specimens are rapidly replaced by mouse stromal cells including fibroblasts, inflammatory cells, blood vessels, and immune cells, and these elements are difficult to introduce in humanized mouse. As reported, expression profiling based on species-specific RNA sequencing of PDXs provides a unique opportunity to distinguish mouse stroma-derived transcripts from human cancer cell-derived transcripts without physically separating the two components prior to RNA extraction (87). Novel approaches, such as short-term primary cultures or organoids, are being developed and are expected to be applied to preclinical screening studies (24). Clinical trials using PDOs are ongoing (25), and PDX-derived organoids are useful for drug screening.

## Conclusions

Integrated analyses of the genome, epigenome, and transcriptome are yielding biological insights with potential therapeutic relevance in PDAC. Genome-based therapies have led to paradigm-changing treatments for other cancers and have dramatically improved survival and cure rates. Therapeutic strategies based on gene alterations in cancer cells, including HRD and MMR-D/MSI-H, have improved the survival of PDAC patients. In the You Know Tumor trial, the OS was significantly longer in patients who received a tailored therapy than in those without an actionable molecular change. However, this remains an unfulfilled promise in PDAC because of the limited number of patients and the rapidity of disease progression. The rapid analysis of genetic mutations using liquid biopsy and new biomarkers, such as BRCA-ness, signature3, and higher TMB, may allow more patients to be recruited for personalized therapy. In addition, the difficulty of drug delivery through the stromal barrier in tumors contributes to high resistance to available chemotherapeutic agents, and therapeutic strategies targeting stromal components have failed due to their complexity. In addition to genomic subtypes, transcriptomic analyses revealed the associations of cancer cell and CAF subtypes with immune cell components, providing biological insights relevant to the treatment of PDAC. Furthermore, phenotypic characterization of individualized models such as PDXs and PDOs will provide additional information for selecting tailored therapies for PDAC patients. Individualized PDXs have the potential to identify effective therapies; however, they have significant limitations, including long lead times and the need for large amounts of tumor tissue for testing. The PDO platform can be exploited for genomic and functional studies even during chemotherapy, with the possibility of selecting sensitive therapeutic agents after acquisition of chemoresistance. New approaches such as co-culture of PDOs with stromal components and humanized PDX may bridge the gap between cancer genetics and patient clinical trials and allow for personalized therapy, although further studies are needed to validate this approach. A multi-parameter approach that combines genome-based medicine with drug screening using individualized models will be key for precision medicine.

## Author Contributions

KM wrote the paper and prepared figures. HN revised manuscript and prepared figures. KK supervised the entire project. All authors contributed to the article and approved the submitted version.

## Funding

This research was supported by Takeda Science Foundation, Ichiro Kanehara Foundation, MSD Life Science Foundation, The Naito Foundation, Kobayashi Foundation for Cancer Research, and Life Science Foundation of Japan.

## Conflict of Interest

The authors declare that the research was conducted in the absence of any commercial or financial relationships that could be construed as a potential conflict of interest.
